# The Role of Orthotactics in Language Switching: An ERP Investigation Using Masked Language Priming

**DOI:** 10.3390/brainsci10010022

**Published:** 2019-12-31

**Authors:** Aina Casaponsa, Guillaume Thierry, Jon Andoni Duñabeitia

**Affiliations:** 1 Department of Linguistics and English Language, Lancaster University, Lancaster LA1 4YL, UK; 2 School of Psychology, Bangor University, Bangor LL57 2AS, UK; g.thierry@bangor.ac.uk; 3 Centro de Ciencia Cognitiva C3, Universidad Nebrija, 28015 Madrid, Spain; jdunabeitia@nebrija.es; 4 Department of Language and Culture, The Arctic University of Norway, 9019 Tromsø, Norway

**Keywords:** bilingualism, event-related potentials, orthotactics, language detection, masked priming

## Abstract

It is commonly accepted that bilinguals access lexical representations from their two languages during language comprehension, even when they operate in a single language context. Language detection mechanisms are, thus, hypothesized to operate after the stage of lexical access during visual word recognition. However, recent studies showed reduced cross-language activation when sub-lexical properties of words are specific to one of the bilingual’s two languages, hinting at the fact that language selection may start before the stage of lexical access. Here, we tested highly fluent Spanish–Basque and Spanish–English bilinguals in a masked language priming paradigm in which first language (L1) target words are primed by unconsciously perceived L1 or second language (L2) words. Critically, L2 primes were either orthotactically legal or illegal in L1. Results showed automatic language detection effects only for orthotactically marked L2 primes and within the timeframe of the N250, an index of sub-lexical-to-lexical integration. Marked L2 primes also affected the processing of L1 targets at the stage of conceptual processing, but only in bilinguals whose languages are transparent. We conclude that automatic and unconscious language detection mechanisms can operate at sub-lexical levels of processing. In the absence of sub-lexical language cues, unconsciously perceived primes in the irrelevant language might not automatically trigger post-lexical language identification, thereby resulting in the lack of observable language switching effects.

## 1. Introduction

Visual word recognition is an interactive, complex, and orchestrated process where different (sub)lexical units compete for selection, modulating the speed and accuracy with which we are able to efficiently access meaning. In bilinguals, the process of recognizing a given word also entails cross-language competition and selection mechanisms [1,2,3] To efficiently map lexical forms onto semantic representations, bilinguals may need to select the language in which the word is written, a process also known as language tagging. For instance, the word *pie* does not have the same meaning for a native speaker of English and a native speaker of Spanish (*pie* means “foot” in Spanish). In this case, Spanish–English bilinguals might need to select the language to which the word belongs in order to disambiguate between the two meanings and the correct phonological representations. 

Understanding the mechanisms involved in language detection is the key to disentangling whether bilingual word processing is language-selective or not. It is now commonly accepted that, when bilinguals encounter words in an ambiguous language context (e.g., a sign on the street in a bilingual community), word recognition is characterized by parallel access to representations of both their languages, and that language detection mechanisms intervene only at a post-lexical processing stage (see the Bilingual Interactive Activation + model, BIA+, proposed by Dijkstra and van Heuven, [4]). Indeed, there is extensive empirical evidence showing that lexical representations of the two languages compete for selection in the bilingual brain (e.g., References [5,6,7,8,9,10,11,12]), where both languages are co-activated during comprehension, even when bilinguals find themselves in a single language context (e.g., References [1,3,5,13,14,15,16,17,18,19,20,21,22,23,24]), and where language information is accessed after the activation of lexical representations (e.g., References [4,11,15,16,17,18,19,20,21,22,23,24,25,26,27,28,29]). It is, therefore, suggested that language identification either relates to top-down modulations from language nodes feeding information back to lexical units [30] or to the operation of a task decision system directly receiving information from the language nodes during word identification [4]. In either case, an English–Spanish bilingual would recognize that the word *happy,* for instance, is a word of English once its (complete) lexical representation is activated.

However, even in ambiguous language contexts, salient visual characteristics of words can afford language identity information before lexical access takes place. When bilinguals encounter words from different scripts, for example, specific features of letters can already trigger language identity resolution. In same-script bilinguals, language identity information can be visually extracted from single graphemes or sub-lexical orthographic patterns (i.e., orthotactics). The word *happy*, for instance, cannot be Spanish because the bigram “pp” does not exist in the Spanish vocabulary. Van Kesteren et al. [31] proposed an extension of the BIA+ model enabling the resolution of language identity at pre-lexical stages of processing. According to the authors, language-specific sub-lexical or feature-level information can be read out by sub-lexical language nodes susceptible to feeding information to the task decision system. Thus, the BIA+ extended model predicts that pre-lexical language-specific information can speed up language attribution, without affecting cross-language lexical activation. However, although the BIA+ extended model effectively accounts for the mechanisms by which word forms are associated with a given language at a pre-lexical stage of processing, it does not account for language-selective effects arising within an integrated lexicon (e.g., [31,32,33,34,35,36,37,38]). 

Recent studies demonstrated that pre-lexical language-specific cues can trigger language selective access and/or reduce cross-language lexical activation. At the feature level, Dubey et al. [38] showed language-selective access for L2 words only when they were incongruent with first language (L1) script. They tested fluent Hindi–English bilinguals in a masked translation priming paradigm. Hindi words could either be presented in the standard Devangari script or in the Roman alphabet. Whilst script differences did not affect L1-to-second language (L2) masked priming translations effects, they did constrain L2-to-L1 translation priming. Hindi–English bilinguals only showed L2-to-L1 priming when L1 targets and primes were in the same script (i.e., the Roman alphabet). These results suggest that bilinguals are highly sensitive to L1 violations and that language-selective mechanisms at the feature level can reduce cross-language lexical activation. Similarly, Oganian et al. [37] showed language-selective effects for same-script bilinguals based on language-specific sub-lexical information. They tested German–English bilinguals in a forced language decision task on pseudowords. Pseudowords could either be marked (i.e., comprise language-specific bigram combinations) or unmarked (i.e., only comprise language-common bigram combinations) at a sub-lexical level but also lexically biased toward one of the two languages (i.e., have more neighbors in one language than the other). Whilst L1-marked pseudowords showed increased inhibitory effects (i.e., slower reaction times) with an increase in L2 neighbors, L2-marked pseudowords did not show any reliable effect of L1 neighborhood density. Both of the abovementioned studies, thus, suggest that fluent bilinguals are highly sensitive to violations of L1 properties at a pre-lexical level and that this can constrain cross-language lexical activation.

Casaponsa and Duñabeitia [33] further reported language-selective effects in a masked translation priming paradigm with same-script bilinguals. Spanish–Basque balanced bilinguals performing a lexical decision task on Spanish words showed a translation priming effect only for Basque-masked primes that were orthotactically legal in Spanish. In an attempt to better characterize the mechanisms underlaying automatic language identification, Casaponsa et al. [35] tested Spanish–Basque bilinguals and Spanish monolinguals in a masked language priming paradigm in combination with event-related potentials (ERPs). Whilst all groups showed automatic language identification effects for unconsciously perceived marked Basque words and within the time frames of the N250 (an index of sub-lexical-to-lexical mapping; see Grainger and Holcomb, [39]) and the N400 (an index of lexical to semantic integration), the bilingual group was blind to masked language switching effects for unmarked Basque primes. These results suggest that sub-lexical language cues can speed up language selection mechanisms (see also References [34,40,41,42]), and that this in turn might affect cross-language lexical activation [23,32,33,43,44,45].

The present study was set out to further characterize automatic language detection processes guided by language-specific sub-lexical information in two groups of bilinguals with different L2. We tested Spanish–Basque and Spanish–English bilinguals in a go/no-go task using a masked language switching paradigm with visual presentation of words in combination with ERPs. Basque and Spanish are orthographically transparent, meaning that they entail a nearly one-to-one correspondence between letters and sounds. In contrast, English is an opaque language and, thus, efficient semantic access relies more on whole-word recognition and activation of phonological representations at a lexical level. Although previous studies investigated the role of script and sub-lexical information in language identification and cross-language activation, little is known about the role of orthotactics in automatic language detection mechanisms and their impact on subsequent orthographic and/or phonological whole-word representations. To investigate the automaticity of language detection mechanisms, we presented bilingual participants with L1 Spanish target words preceded by unconsciously perceived word primes either in L1 Spanish or in their other language (L2 Basque or English). Critically, L2 primes could either be orthographically marked (i.e., contain illegal bigram combinations in L1) or unmarked (i.e., orthographically legal also in L1). Furthermore, and different from our previous study [35], L1 and L2 primes were translation equivalents so that the conceptual distance between primes and targets was controlled across languages. Participants were asked to detect words referring to an animal (go trials, ~10%, not analyzed) so as to make sure that they processed the meaning of L1 words in the analyzed no-go trials. To minimize effects related to conscious language identification mechanisms, all instructions were given in L1, and none of the bilinguals were aware of the presence of L2 stimuli during the session. 

According to the dual-route model of language identification proposed by van Kesteren et al. [31], language information at sub-lexical and lexical levels is picked-up by sub-lexical and lexical language nodes, respectively. Language information can then be read out by the task-decision system, without affecting word identification processes. That is, sub-lexical language-specific information should not affect cross-language sub-lexical-to-lexical integration, nor should language detection at the lexical levels affect cross-language lexical activation or lexical–semantic integration. However, our previous results seem to suggest that, whilst lexical language information fails to affect word identification, sub-lexical language information does have an impact on subsequent word processing [35]. Therefore, according to the theoretical time-course of visual word recognition in masked priming paradigms (see Bi-modal Interactive Activation Model, BIAM, [39]), we expected L2 marked primes to elicit a N250 modulation, reflecting the process of mapping sub-lexical information onto word form representations in both groups of bilinguals, that is, whether their L2 is transparent (Basque) or opaque (English). Hence, L2 marked words should automatically activate sub-lexical language nodes and, in line with our previous results [35], this could modulate sub-lexical-to-lexical integration and affect subsequent lexical–semantic integration indexed by N400 effects. However, considering the bi-modal route to semantics (i.e., orthographic or phonological) posited in the BIA+ [4] and the BIAM [39], if orthographic rules selectively constrain orthographic whole-word representations, then Spanish–English bilinguals might show intact lexical–semantic integration for L2 marked words. Furthermore, consistent with previous studies and the prediction of the BIA+ and BIA+ extended models, we did not expect automatic post-lexical language identification effects to emerge (in the N400 range) in an L1 context when unconsciously perceived L2 primes are unmarked (language-common orthography). 

## 2. Experiment 1

### 2.1. Materials and Methods

#### 2.1.1. Participants

Eighteen right-handed highly proficient Spanish–Basque bilinguals from the Basque Country participated in this experiment (11 women; mean age = 22.2, SD = 4.1). All participants were native Spanish speakers fluent in Basque (Table 1). None of the participants reported neurological or psychiatric disorders, all participants had normal or corrected-to-normal vision, and they participated voluntarily in this experiment in exchange for monetary compensation. Prior to the experimental session, all participants gave their written informed consent in accordance with guidelines approved by the Ethics and Research Committees of the Basque Center on Cognition, Brain, and Language. The study was also performed in accordance with the ethical standards set in the Declaration of Helsinki.

#### 2.1.2. Stimuli

We selected 280 Spanish (L1) words to serve as targets (e.g., *casa*—“house”), from the Spanish B-Pal database [46]. Primes were either unrelated Basque (L2) words (280 items) or their translations in Spanish (280 items), selected from the E-Hitz database [47] and B-Pal, respectively. None of the prime or target words were cognates in order to avoid confusion between languages. Mean length-corrected Levenshtein distance (cLD) (calculated from the number of deletions, substitutions, and additions between pairs of words divided by the length of the longest word; 1 indicates full overlap between pairs of words, and 0 indicates no orthographic overlap) for marked translation equivalents was 0.13 (SD = 0.11), and mean cLD for unmarked translation equivalents was 0.14 (SD = 0.10). Prime–target combinations were created avoiding semantic and orthographic overlap (i.e., all prime–target word pairs were unrelated), and primes and target were either in the same language (both in L1) or in a different language (primes in L2 and targets in L1). Critically, the L2 Basque prime words were split into two groups: 140 contained bigrams illegal in Spanish, and the other 140 contained bigrams valid in Spanish. Thus, half of the Spanish targets were preceded by either (1) an unrelated Basque word containing bigrams illegal in the L1 (e.g., *txakur*—“dog”, with the bigram “tx” being an illegal bigram in Spanish), or by (2) the translation equivalent in Spanish of the Basque prime (e.g., *perro*—“dog”). The other half of the Spanish targets were preceded by either (1) an unrelated Basque word orthographically legal in Spanish (e.g., *mendi—*“hill”), or by (2) the translation in Spanish of the Basque prime (e.g., *monte—*“hill”). Stimuli were matched across conditions for word frequency, number of letters, number of orthographic neighbors, age of acquisition, and concreteness (Table 2). Furthermore, we also matched primes and targets for cLD (L1 marked control: mean = 0.14, SD = 0.11; L2 marked: mean = 0.12, SD = 0.10; L1 unmarked control: mean = 0.13, SD = 0.11; L2 unmarked: mean = 0.13, SD = 0.10). Note that the idiosyncratic distributional properties of individual letters and their combinations do not match across languages. Hence, Basque primes had overall lower mean bigram frequency in Spanish than Spanish primes. Importantly, we matched mean bigram frequency across the two critical conditions (marked vs. unmarked). Using a counterbalanced (Latin square) design, we created two lists so that each target word appeared only once in each list, but every time in a different priming condition. This resulted in 70 trials per condition in each list, which served as no-go trials in the semantic categorization task. Participants were randomly assigned to each list. An additional set of 35 animal names in Spanish were selected as go trials (10%), all preceded by unrelated masked primes. We also included a prime visibility test in order to provide an estimate of the level of visibility of the prime stimuli. The same set of animal names was used as masked primes followed by unrelated Spanish targets for the prime visibility test. See Table A1 (Appendix A) for the full set of stimuli used in this experiment.

#### 2.1.3. Procedure

Participants were tested individually in a quiet room. Visual stimuli were presented using Presentation software (Version 4.6, Neurobehavioral systems, Inc., Albany, OR, USA) on a 15” cathode-ray tube (CRT) monitor set to a refresh rate of 60 Hz (which allows for 16.67 ms vertical retraces). Stimuli were displayed at high contrast in white letters on a black background. In each trial, a forward mask consisting of a row of hashmarks (#’s) was presented for 500 ms. Next, the prime was presented in 25 pt lowercase Courier New and stayed on the screen for ~50 ms (three refresh rate cycles). The prime was immediately followed by the presentation of the target stimulus in 25 pt uppercase Courier New. The target remained on the screen for 500 ms. The inter-trial interval varied randomly between 900 and 1100 ms. After this interval, an asterisk was presented for 1000 ms in order to allow for participants’ blinks. Masks, primes, and targets were presented in the center of the screen. In order to ensure participants’ attention whilst passively reading the critical words, a go/no-go semantic categorization task was imposed where the critical stimuli did not require an overt response. Participants were instructed to press the space bar of a keyboard whenever they detected an animal name on the screen. They were not informed of the presence of the primes. Trial presentation order was randomized across participants. Each participant received a total of 20 practice trials (representative of the conditions in the critical trials) prior to the 280 experimental trials. Task instructions (and interactions with the participants) were given in their L1 (Spanish). The experimental session lasted for approximately 20 min (excluding participants’ preparation).

#### 2.1.4. Electroencephalogram (EEG) Recording Procedure

The electroencephalogram was recorded from 27 electrodes (plus ground) held in place on the scalp by an elastic cap (ElectroCap International, Eaton, USA, 10-10 system). Eye movements and blinks were monitored with four further electrodes providing bipolar recordings of the horizontal (Heog−, Heog+) and vertical (Veog−, Veog+) electrooculogram (EOG). Another two electrodes were attached over the right mastoid bone (online reference) and over the left mastoid bone. All EEG electrode impedances were maintained below 5 kΩ (impedance for eye electrodes was less than 10 kΩ). The EEG signal was sampled continuously throughout the experiment at 250 Hz and digitally offline re-referenced to linked mastoids. 

#### 2.1.5. Data Analysis

Ocular artefacts were corrected using independent component analysis (ICA). Based on previous literature, the ICA algorithm used was Infomax (gradient) restricted biased. A high-pass filter of 0.01 Hz was applied before the ICA procedure, and a low-pass filter of 30 Hz was applied after ICA. Averaged ERPs time-locked to target onset were computed offline from trials free of ocular and muscular artefacts (85% of the data; rejected trials were equally distributed across conditions). Baseline correction was performed using the averaged EEG activity in the 100 ms preceding the onset of the target stimuli. 

ERPs were quantified by taking the mean amplitude of each participant and electrode in three temporal epochs corresponding to two key components: The N250 (covering a time-window between 200 and 300 ms post-target onset) and the N400, represented by an early time-window between 350 and 500 ms (N400s which indexes lexical-semantic integration; see Reference [39]) and a late time-window between 500 and 600 (N400c, which indexes concept-to-concept mapping). These two components of interest correspond with those reported in earlier studies on the same topic using a similar procedure [11,25,26,48,49,50]. ERP mean amplitudes in each time-window were analyzed separately using repeated measures analyses of variance (ANOVAs). Out of the 27 electrodes, 21 were used for the analysis, distinguishing three levels of region comprising seven adjacent electrodes each: anterior (Fp1|Fp2|F7|F3|Fz|F4|F8), central (FC5|FC1|FC2|FC6|C3|Cz|C4|), and posterior (CP5|CP1|CP2|CP6|P3|Pz|P4). Two other factors associated with the design were included in the analyses: language (switch, non-switch) and markedness (marked, unmarked). Greenhouse–Geisser correction was applied for departure from sphericity [51]. Greenhouse–Geisser epsilon value (ε) is provided only when different from 1, indicating a violation of the assumption of sphericity, and the corrected *p*-value is, therefore, reported. Effect sizes were estimated using the partial eta-squared coefficient *η^2^_p_* [52,53].

### 2.2. Results

#### 2.2.1. Behavioral Results

Bilingual participants correctly categorized 92.18% (SD = 4.01) of the animal names in Spanish when these words were presented as targets (percentage of false alarms: 0.59%, SD = 0.45; percentage of accuracy in the prime visibility test: 0.47%, SD = 2.18). None of the participants reported consciously perceiving the animal names (or any other word) when presented as primes, confirming that participants were unaware of the existence and nature of the masked primes.

#### 2.2.2. ERPs Results 

**Figure 1:**
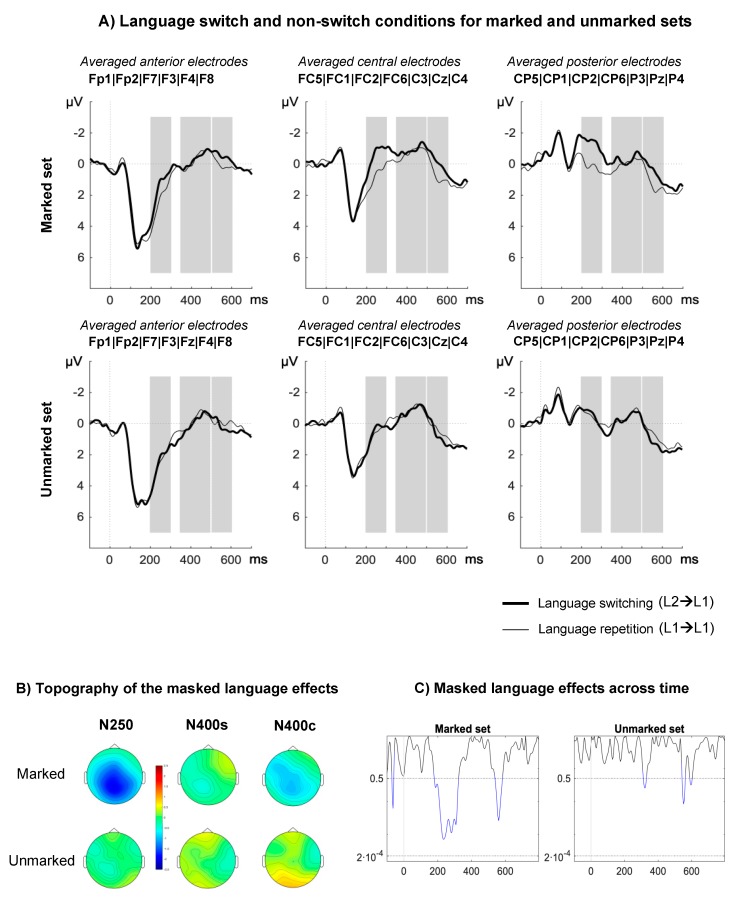
Event-related potential (ERP) results in Experiment 1: highly proficient Basque–Spanish bilinguals. (**A**) ERPs associated with the switch (thick lines) and non-switch conditions (thin lines) in the marked (upper) and unmarked (lower) sets. Time-windows of analysis are marked in gray. (**B**) Topographical distribution of the language switch effects for marked and unmarked priming conditions in terms of amplitude differences between the unrelated Basque and unrelated Spanish primes. (**C**) Time-course of *p*-values for the language switch effect in marked and unmarked sets. The *p*-values are calculated from individual *t*-tests for every data point (every 4 ms; 225 comparisons in total) and collapsed across all electrodes. Blue lines indicate *p*-values >0.05. The Bonferroni correction threshold is marked on the graph (α = 0.05/255; *p* = 0.0002).

**N250.** Results revealed a main effect of language (F(1,17) = 14.11, *p* < 0.01, *η^2^_p_* = 0.45), showing that switch trials elicited more negative-going waveforms than non-switch trials. The main effect of markedness was significant (F(1,17) = 5.73, *p* = 0.028, *η^2^_p_* = 0.25), showing that marked primes produced greater negativities than unmarked primes. Critically, the interaction between language and markedness was significant (F(1,17) = 10.67 *p* ≤ 0.01, *η^2^_p_* = 0.38). Planned comparisons showed significant masked language switching effects for L2 marked primes (F(1,17) = 16.71, *p* = 0.001, *η^2^_p_* = 0.50), but not for L2 unmarked primes (F(1,17) = 0.52, *p* = 0.48, *η^2^_p_* = 0.03; see Figure 1). Language and markedness interacted with region ((F(2,34) = 4.29, *p* = 0.05, *η^2^_p_* = 0.20, ε = 0.59) and (F(2,34) = 7.08, *p* = 0.01, *η^2^_p_* = 0.29, ε = 0.62), respectively), and a three-way interaction between these factors was also found (F(2,34) = 5.50, *p* = 0.02, *η^2^_p_* = 0.24, ε = 0.61). Post hoc comparisons showed that the magnitude of the switching effect increased at central–posterior regions for marked primes (anterior–central: t(17) = 3.72, *p* < 0.01; central–posterior: t(11) = 1.40, *p* = 0.18), whilst it was constantly distributed for unmarked primes (all *p* > 0.72).

**N400s.** No relevant main effects or interactions were found significant in this time-window (*p* > 0.53). 

**N400c.** We found an interaction between language and markedness (F(1,17) = 10.26, *p* < 0.01, *η^2^_p_* = 0.38). Planned comparisons showed significant language switching effects only for L2 marked primes (L2 marked: F(1,17) = 4.98, *p* = 0.04, *η^2^_p_* = 0.23; L2 unmarked: F(1,17) = 2.59, *p* = 0.13, *η^2^_p_* = 0.13). The remaining relevant main effects and interactions did not approach significance (all *p* > 0.21).

## 3. Experiment 2

### 3.1. Materials and Methods

#### 3.1.1. Participants

Eighteen right-handed fluent Spanish–English bilinguals (11 women; mean age: 20.94, SD = 0.87) with normal or corrected-to-normal vision participated in this experiment in exchange for monetary compensation. All of them were proficient in English according to their self-rated proficiency (Table 3). None of the participants reported neurological or psychiatric disorders. Prior to the experimental session, all participants gave their written informed consent in accordance with guidelines approved by the Ethics and Research Committees of the Basque Center on Cognition, Brain, and Language. The study was also performed in accordance with the ethical standards set in the Declaration of Helsinki.

#### 3.1.2. Materials

We selected 304 Spanish (L1) words to serve as targets (e.g., *cuento—*“tale”), from the Spanish B-Pal database [46]. Primes were either unrelated English (L2) words (304 items) or unrelated Spanish (L1) words (304 items) selected from the N-Watch database [54] and B-Pal, respectively. As in Experiment 1, unrelated English and Spanish words (i.e., L1 and L2 primes) were non-cognate translation equivalents (cLD for marked set: mean = 0.20, SD = 0.16; unmarked set: mean = 0.19, SD = 0.16). Experimental conditions and manipulations were exactly the same as in Experiment 1. The 304 Spanish targets were split into two sets matched for word frequency, word length, number of orthographic neighbors, and mean bigram frequency (see Table 4). In one of the sets, targets could be preceded by (1) an unrelated English word containing bigrams illegal in the L1 (e.g., black, with the bigram “ck” not being legal in Spanish), or (2) the Spanish translation of the English prime word (e.g., *negro*). As for the other set of Spanish words, targets could be preceded by (1) an unrelated English word containing bigrams legal in Spanish (e.g., brain), or (2) the Spanish translation of the English word (e.g., *cerebro*). Prime–target overlap was also controlled (cLD for marked sets: L1–L1—mean = 0.12, SD = 0.10; L2–L1—mean = 0.11, SD = 0.09; cLD for unmarked sets: L1–L1—mean = 0.11, SD = 0.11; L2–L1—mean = 0.10, SD = 0.09). Two lists were created and counterbalanced across participants, so that each target word appeared only once in each list, but in a different prime condition every time. Participants were randomly assigned to each list, and priming conditions were evenly distributed across and within lists (304 critical prime–target pair words in each list, with 76 word pairs per condition). All pairs were used as no-go trials in a semantic categorization task, with an additional set of 38 animal names in Spanish serving as targets (12.5%) or go trials. As in Experiment 1, a prime visibility test was embedded in Experiment 2. The full list of stimuli is provided in Table A2 (Appendix A). Procedure, EEG recording, and data analysis were the same as in Experiment 1.

### 3.2. Results

#### 3.2.1. Behavioral Results 

Bilingual participants correctly categorized 93.22% (SD = 3.51) of the animal names when these were presented as targets (percentage of false alarms: 0.59%, SD = 0.45; percentage in the prime visibility test 0.47%, SD = 2.18). None of the participants reported having consciously perceived any animal name (or any other word) presented as a prime, confirming that participants were unaware of the existence and nature of the masked primes.

#### 3.2.2. ERP Results 

**N250.** There was a significant main effect of language on N250 mean amplitude (F(1,17) = 6.58, *p* = 0.02, *η^2^_p_* = 0.28), such that switch trials elicited more negative-going waveforms than non-switch trials. This effect was modulated by markedness (F(1,17) = 4.28, *p* = 0.05, *η^2^_p_* = 0.20). Planned comparison showed that only marked conditions elicited significant masked language switch effects (marked: F(1,17) = 10.30, *p* = 0.005, *η^2^_p_* = 0.38; unmarked: F(1,17) = 0.34, *p* = 0.59, *η^2^_p_* = 0.02). A marginal interaction was also found between markedness and region (F(2,34) = 3.65, *p* = 0.07, *η^2^_p_* = 0.18, ε = 0.59), showing that an effect of markedness was only found at posterior electrodes (anterior: F(1,17) = 0.01, *p* = 0.95, *η^2^_p_* < 0.001; central: F(1,17) = 1.32, *p* = 0.27, *η^2^_p_* = 0.07; posterior: F(1,17) = 4.38, *p* = 0.05, *η^2^_p_* = 0.21). Other main effects and interactions did not approach significance (*p* > 0.21)

**N400w.** The main effect of markedness on N440w mean amplitude was significant (F(1,17) = 7.69, *p* = 0.01., *η^2^_p_* = 0.31), showing that unmarked conditions elicited more negative-going waveforms than marked conditions. Other relevant main effects and interactions did not approach significance (*p* > 0.13).

**N400c.** No relevant main effects or interactions were found in this time-window (all *p* > 0.13).

## 4. Discussion

In this study, we sought to determine whether orthotactic cues can trigger unconscious language identification mechanisms in two groups of bilinguals differing in terms of language pairs and context of language learning. As in previous studies (e.g., References [11,25,35,50]), we focused on modulations of the N250 and N400 components. Masked language switching modulated N250 amplitude as a function of the orthographic regularities of L2 words. As expected, this early language switch effect was only found for those L2 primes (in Basque or English) that contained at least one bigram illegal in L1 (Spanish). Note that language switching effects were not observed in either the N250 or the N400 time-window in the case of unmarked sets. Language switching also modulated N400c amplitude in the case of L2 marked words only, and only in fluent bilinguals whose two languages are transparent (i.e., Spanish and Basque). Although they showed early language switching effects triggered by L1 orthotactic violations, Spanish–English bilinguals did not show modulations in later stages of visual word recognition. 

**Figure 2:**
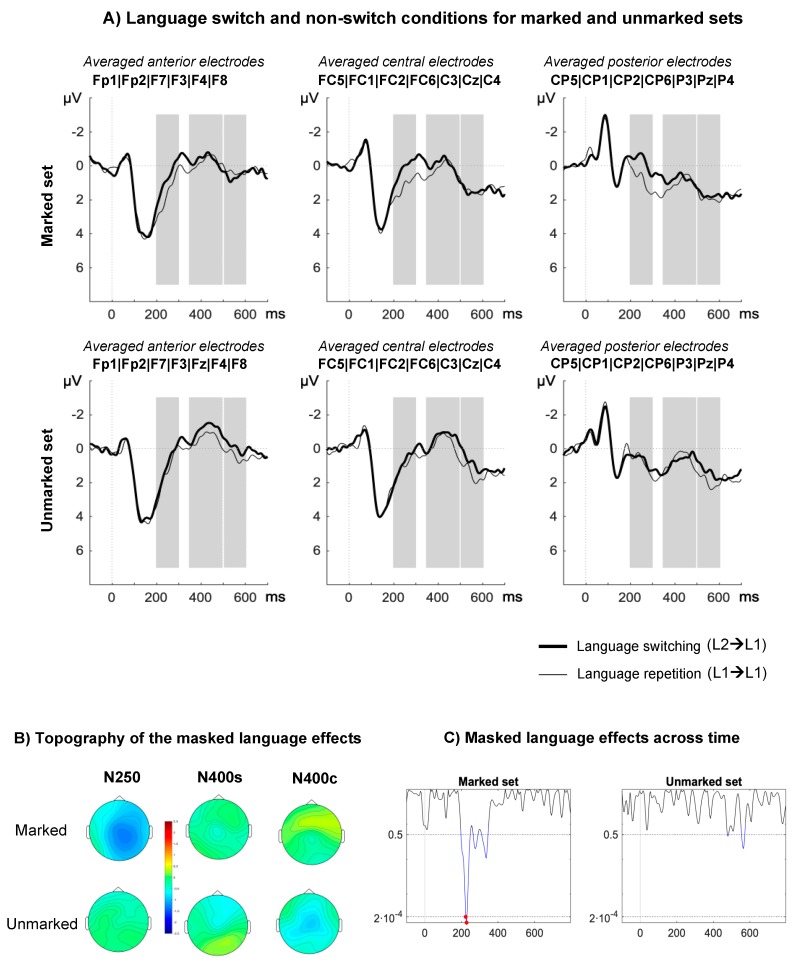
ERP results in Experiment 2: highly proficient Spanish–English bilinguals. (**A**) ERPs associated with the switch (thick lines) and non-switch conditions (thin lines) in the marked (upper) and unmarked (lower) sets. Time-windows of interest are marked in gray. (**B**) Topographical distribution of the language switch effects for marked and unmarked priming conditions in terms of amplitude differences between the unrelated Basque and unrelated Spanish primes. (**C**) Distribution of *p*-values for the language switch effect in marked and unmarked sets. The *p*-values are calculated from individual *t*-tests for every data point (every 4 ms, 225 comparisons in total) and collapsed across all electrodes. Blue lines indicate *p*-values < 0.05. Red lines indicate *p*-values < 0.0002 (Bonferroni corrected significance level; α = 0.05/255; *p* = 0.0002).

**Figure 3:**
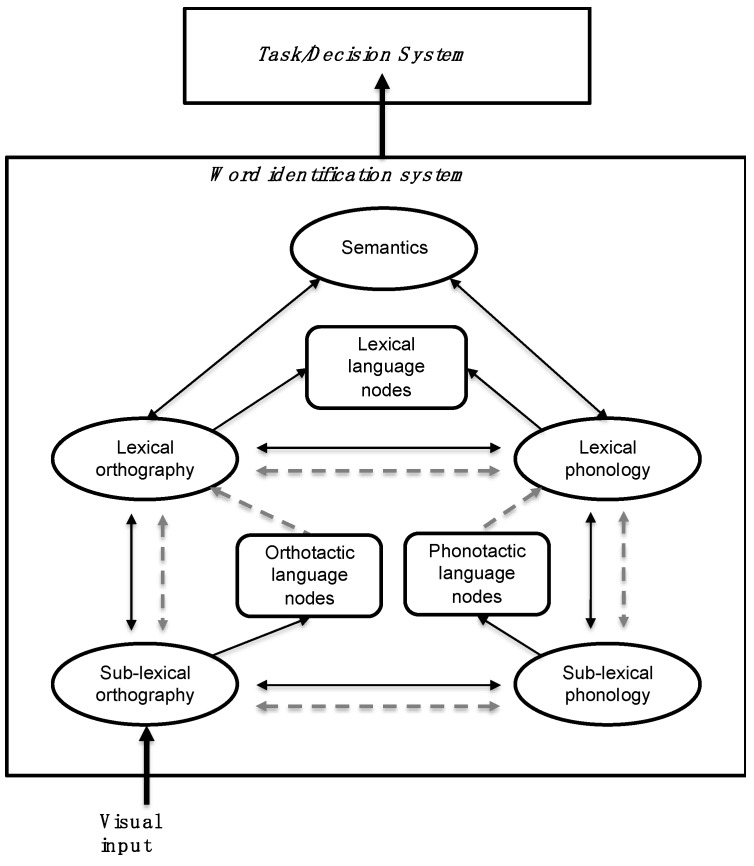
Proposed modification of the Bilingual Interactive Activation + (BIA+) extended model to account for language-selective effects emerging within an integrated lexicon, tentatively labeled BIA+s. We implement the separation of the sub-lexical language node into two different nodes: orthotactic language nodes for the orthographic route to semantics, and phonotactic language node for the phonological route. Each node can receive information from their corresponding sub-lexical units and feed-forward inhibitory links to the lexical orthography (orthotactic language node) or the lexical phonology (phonotactic language node). Dashed lines represent inhibitory connections.

When L2 primes were orthographically marked, we showed a modulation of target processing as early as 200 ms post stimulus onset followed by modulations in the window corresponding to conceptual processing (Figure 1C). This result is consistent with the view that orthotactic patterns can trigger automatic language detection mechanisms at a sub-lexical level as implemented in the BIA+ extended model [31]. However, our results also suggest that sub-lexical language nodes can modulate cross-language lexical activation of whole-word orthographic representations, which the BIA+ extended model cannot currently account for. To account for this effect, the model would need to include an inhibitory link between the sub-lexical language node and the orthographic lexicon. Furthermore, it is unlikely that the sub-lexical language node can modulate access to whole-word phonological representations. We contend that this is why switching from L2 marked English primes to L1 targets does not hamper semantic integration more than when both primes and targets are in L1. Thus, we assume the existence of two separate sub-lexical language nodes (Figure 3) in line with the dual-route account of semantic access implemented in the BIA+ model [4]. Note that the lack of an N400 effect for L2 English marked primes cannot be explained by relatively lower proficiency of participants in their L2. If proficiency in L2 plays a role, differences between experiments could be expected in the opposite direction, since Spanish–English bilinguals, contrary to Spanish–Basque bilinguals, learnt L2 in an academic context and were not daily exposed to their L2. Indeed, greater modulation would be expected for L2 English compared to L1 Spanish primes in the time-window related with semantic integration (N400), since such effects were observed in Spanish participants with no knowledge of L2 (see Reference [35]). Since no such effects were found, proficiency and exposure alone cannot account for our results. Another possibility is that the long-lasting effect registered in the N250 time-window masked effects occurring at a later point (see Figure 1C and Figure 2C). However, this would make N400 effects found in Experiment 1 and in previous studies (e.g., Reference [35]) difficult to account for. Instead, we contend that the difference between experiments relates to the degree of transparency of the languages involved and the similarities across language of grapheme-to-phoneme mapping rules. Given that English has a shallow orthography, participants would rely more on the phonological route to access lexical representations (see BIA+ model [4]), leading to non-selective lexical access. Therefore, even though sub-lexical orthographic cues are detected in the N250 window, these cues are not sufficient to constrain lexical access, which can be achieved through the phonological route.

When L2 primes are not orthographically marked, no ERP amplitude modulation is detected in the N250 or N400 time-windows. That is, Spanish–Basque and Spanish–English bilinguals do not show reliable signs of masked language switching effects for those L2 primes that are orthographically similar to L1. Similar effects were also found in the study by Casaponsa et al. [35], where highly proficient Spanish–Basque bilinguals showed no reliable masked language switching effect for unmarked sets. Note that the authors did find a modulation of target processing for both L2 marked and unmarked primes in both the N250 and N400 time-windows in a group of Spanish monolinguals. We conclude that the lack of a masked language switching effect for unmarked L2 primes results from cross-language lexical activation in fluent bilinguals and, therefore, that lexical and semantic access in highly proficient bilinguals is not affected by unconscious language switching when L2 primes comply with L1 orthotactics. These results are consistent with the predictions of the BIA+ [4] and the BIA+ extended [31] models, whereby lexical-level language identification nodes do not feed information back to the lexicon but rather feed information directly to the task decision system. According to this view, lexical language nodes would not affect cross-language lexical activation by inhibiting the irrelevant language, as previously implied by the structure of the BIA [30], and language identification would only impact participants’ responses when the task (or the language context) requires some degree of language discrimination.

It is worth noting that one of the factors that could not be equated across marked and unmarked sets was mean Spanish bigram frequencies across languages. This measure extracted from the B-Pal, N-Watch, and E-Hitz databases is position- and length-dependent. Even though primes were carefully matched for mean Spanish bigram frequency across the two language sets, the mean Spanish bigram frequency of Spanish words was overall slightly higher than that of Basque and English words (*p* < 0.05). This difference naturally relates to the inherent difference in bigram distributions between languages and the fact that L1 and L2 primes were translations equivalents one each other. Therefore, the Basque and English prime sets were orthographically infrequent in Spanish, but this difference did not lead to any measurable N250 modulation in the case of the unmarked sets. Therefore, automatic language identification seems to rely on violations of L1 orthotactics rather than subtle sub-lexical statistical probabilities (see also Reference [37]).

Since both the BIA+ [4] and the BIA+ extended [31] models cannot account for the current results or results from our previous studies showing reduced cross-language effects stemming from sub-lexical level [33,35], we propose a modification of BIA+ extended as follows: firstly, we propose to implement separate orthographic and phonological sub-lexical language nodes in a revised version of the model tentatively named BIA+s (Figure 3); secondly, we implement inhibitory links between orthographic and phonological sub-lexical language nodes and their corresponding lexical forms to allow language-selective effects to emerge [33,35,37]. 

### 4.1. Orthotactic, Phonotactic, and Feature Language Nodes

We propose that the suggested orthographic and phonological language nodes at the sub-lexical level are sensitive to the statistical regularities of their processing route (either orthographic or phonological) and, thus, that orthotactic patterns will be detected by the orthographic sub-lexical node, whilst phonotactic patterns will be detected by the phonological sub-lexical node. 

It is worth noting that, whilst orthotactic patterns are visually salient during visual word recognition, phonotactic patterns rely upon the participant’s ability to access L2 phonological representations nonexistent in their L1. For instance, Spanish–English bilinguals might not correctly convert the word “ham” into a phonologically marked English word (i.e., Spanish phonotactics do not include the sound /h/). Instead, they might convert the grapheme “h” (mute/no sound in Spanish) into to the closest phonemical category in Spanish, /x/ (corresponding to the letter “j”). Hence, we suggest that the phonotactic language node might be specifically sensitive to auditory word recognition in non-balanced bilinguals. 

The detection of orthotactic patterns is influenced by participants’ sensitivity to statistical regularities within languages. So far, the evidence from previous studies suggests that language detection mechanisms are highly sensitive to violations of sub-lexical orthographic regularities of the native language [33,34,35,37,40,41,55], but not of the second language [37,38,40]. In other words, detecting L1 marked words is more difficult than detecting L2 marked words. This is probably because, to detect L1 marked words, bilinguals not only need to be highly proficient in L2, but they also need to internalize the patterns that distinguish their L1 from their L2 (L1 specific vs. L1 common). Further studies exploring literacy balanced bilinguals, or L1 attrited bilinguals are needed in order to test whether sensitivity to L2 orthotactic violations in L1 words increases with L2 reading exposure. Nonetheless, based on current evidence, orthotactic language nodes appear particularly sensitive to regularities of the most frequent language (L1). 

### 4.2. Sub-Lexical Language Nodes and Lexical Access

In BIA+s, orthotactic patterns can constrain cross-language lexical activation via inhibitory links between orthotactic language nodes and orthographic word forms, whilst access to phonological lexical forms is unaffected. In cases where orthographic-to-phonological conversion is similar between languages (e.g., in the case of Spanish–Basque bilinguals) and predominantly based on one-to-one letter–sound correspondence (i.e., in the case of transparent languages), reduced cross-language activation of orthographic lexical representations should impact semantic and conceptual access. For instance, in an L1 Spanish context, unconsciously perceived L2 Basque marked primes would trigger sub-lexical language detection mechanisms which will, in turn, inhibit L1 orthographic lexical representations. L1 target processing would then require the activation of L1 lexical forms previously inhibited by L2 marked primes, delaying L1 lexical selection and subsequent semantic and conceptual processing. This scenario would explain the N400 modulations observed for L1 targets in Experiment 1, which are likely to be the result delayed access to conceptual representations for L2 marked primes due to reduced cross-language orthographic lexical activation. In cases where one or both of the two languages are opaque (e.g., English), semantic and conceptual access would be predominantly mediated by the phonological route. This is how the BIA+s could lead to the expectation that the inhibition of orthographic lexical representations by the orthotactic language node would have little impact on semantic and conceptual access, since this would be mediated by cross-language lexical activation of phonological word forms. In the case of languages with different scripts, cross-language lexical activation is also thought to derive from phonological lexical representations. For instance, Wu and Thierry [18] showed that Chinese–English bilinguals automatically activate representations form their native language (Chinese) when reading in (or listening to) English. These authors found priming effects for L1 hidden sound repetition, but not for hidden orthographic L1 repetition. Thus, bi-scriptal readers would automatically activate cross-language phonological lexical forms, rather than orthographic word forms. The lack of N400 modulations for L2 English marked sets in Experiment 2 corroborates this view. Furthermore, in an L1 Spanish context, the presence of unconsciously perceived L2 English marked primes would automatically activate the orthotactic language nodes inhibiting L1 Spanish orthographic lexical representations, in turn affecting sub-lexical-to-lexical mapping of whole orthographic word forms indexed by modulations of N250 mean amplitudes (see Figure 2A,B). Phonological lexical access would remain available, allowing successful semantic and conceptual access, and explaining the lack of N400 modulation for L2 marked sets in English.

## 5. Conclusions

In sum, by studying fluent Spanish–Basque and Spanish–English bilinguals engaged in a semantic categorization task within a masked language switching paradigm, we were able to show that sub-lexical orthographic information plays a significant role in language detection. Based on the markedness effects reported here, we make suggestions regarding improvements of the BIA+ extended model to allow for language-selective effects to emerge within an integrated lexicon. 

## Figures and Tables

**Table 1:** Experiment 1: Mean levels of first language (L1; Spanish) and second language (L2; Basque) language proficiency derived from participants’ self-ratings (range: 0 = none, 10 = native-like). Standard deviations are provided within parentheses.

Language Proficiency	Spanish	Basque
Speaking	9.78 (0.43)	7.88 (1.36)
Understand	9.78 (0.43)	8.50 (1.20)
Writing	9.78 (0.54)	7.22 (1.53)
Reading	9.83 (0.38)	8.50 (1.24)
General self-perception	9.61 (0.61)	7.66 (0.97)

**Table 2:** Experiment 1: Mean values for each sub-lexical, lexical, and semantic factor in L1 (Spanish) and L2 (Basque) split by condition. Standard deviations are provided within parentheses.

	Prime Words				Target Words	
	Basque		Spanish		Spanish	
	Marked	Unmarked	Marked Control	Unmarked Control	Marked	Unmarked
Word frequency	36.50 (74.78)	33.91 (58.77)	28.53 (36.29)	29.87 (30.47)	41.14 (47.53)	38.66 (44.66)
Word length	7.55 (1.86)	7.37 (2.22)	7.46 (1.91)	7.30 (2.17)	6.66 (1.62)	6.45 (1.69)
Number of orthographic neighbors	1.02 (1.77)	1.26 (2.08)	1.35 (2.52)	1.68 (3.27)	2.42 (4.00)	2.53 (3.94)
Age of acquisition	3.27 (0.47)	3.24 (0.50)	3.17 (0.53)	3.16 (0.56)	3.12 (0.57)	3.09 (0.60)
Word concreteness	4.08 (0.83)	3.99 (0.86)	4.01 (0.85)	3.94 (0.94)	4.05 (0.86)	4.06 (0.91)
Spanish bigram frequency	1.30 “ (0.44)	1.31 “ (0.43)	2.52 (0.29)	2.49 (0.29)		
Basque bigram frequency	2.06 (0.27)	2.04 (0.24)				
Number of Spanish-implausible bigrams	1.16 * (0.45)	0 (0)				

**Note**: Asterisk indicates significant statistical differences between marked and unmarked conditions within sets (Basque or Spanish). Quotation marks denote significant differences between Basque and Spanish words within sets (Marked or Unmarked).

**Table 3:** Experiment 2: Mean levels of Spanish and English language proficiency calculated by participant’s self-perceived ratings. Standard deviations are provided within parentheses.

Language Proficiency	Spanish	English
Speaking	9.78 (0.55)	7.33 (1.08)
Understand	9.83 (0.38)	7.89 (0.96)
Writing	9.56 (0.70)	7.44 (0.92)
Reading	10 (0.00)	8.06 (1.25)
General self-perception	9.83 (0.38)	7.82 (0.88)

**Table 4:** Experiment 2: Mean values for each factor in Spanish and English prime words for each condition, marked and unmarked. Standard deviations are provided within parentheses.

	Prime Words			Target Words		
	English		Spanish	Spanish		
	Marked	Unmarked	Marked Control	Unmarked Control	Marked	Unmarked
Word frequency	33.98 (41.81)	41.70 (60.37)	29.68 (38.19)	34.80 (49.48)	41.01 (33.04)	41.72 (33.36)
Word length	6.75 (1.73)	6.52 (2.04)	6.99 (1.83)	6.80 (1.84)	6.90 (1.91)	6.41 (1.57)
Number of orthographic neighbors	1.66 (2.69)	1.99 (3.15)	1.95 (3.39)	2.39 (3.46)	2.17 (3.34)	2.63 (3.91)
Spanish bigram frequency	1.30 “ (0.52)	1.38 “ (0.47)	2.50 (0.30)	2.54 (0.31)	2.51 (0.33)	2.55 (0.33)
English bigram frequency	2.52 (0.36)	2.55 (0.39				
Number of Spanish-implausible bigrams	1.11 * (0.39)	0 (0)				

Frequency ratings were obtained from the N-Watch and B-Pal databases. The critical factor number of Spanish bigram frequency refers to the number of bigrams from the English words that are not legal in Spanish according to the Spanish LEXESP corpus. An asterisk indicates significant statistical differences between marked and unmarked conditions within sets (Basque or Spanish). Quotation marks denote significant differences between Basque and Spanish words within sets (marked or unmarked). The rest of comparisons (primes within and across sets, and prime–target relationships) were non-significant (all *p* > 0.35).

## Appendix A

**Table A1:** Stimuli used in Experiment 1 using the masked language switching priming paradigm with highly proficient Spanish–Basque bilinguals.

Prime Words				Target Words	
Basque		Spanish		Spanish	
Marked	Unmarked	Marked Controls	Unmarked Controls	Marked Set	Unmarked Set
zuku	ahur	jugo	palma	camarero	vuelo
hozkada	ontasun	bocado	bondad	salud	quiebra
kode	amorru	clave	rabia	suelo	parado
nazka	garaipen	asco	triunfo	enfermera	despido
txanda	sorgin	turno	bruja	desierto	membrana
txano	arrazoi	gorro	motivo	habla	árbol
txartel	zaindari	tarjeta	guardián	problema	rigor
umetoki	laburpen	útero	resumen	vertiente	cuchillo
altxor	aurrerabide	tesoro	progreso	facilidad	convenio
beroki	xehetasun	abrigo	detalle	esposa	libertad
etsai	apaltasun	enemigo	humildad	jardín	alumno
etxola	neurri	cabaña	medida	pito	hijo
hauts	barealdi	polvo	calma	blasfemia	sequía
irrits	sudur	pasión	nariz	cabo	oscuridad
jainko	mingostasun	dios	amargura	candidato	excusa
kopeta	igorle	frente	emisor	ganador	prudencia
kotoi	erdi	algodón	mitad	aula	energía
ostiko	itun	patada	alianza	difusión	vuelta
sukar	kirol	fiebre	deporte	hito	asesinato
txoko	isiltasun	rincón	silencio	vara	venganza
akats	gezur	defecto	mentira	concejal	intestino
amets	ipurdi	fantasía	culo	saludo	altura
arratsalde	zabaltasun	tarde	amplitud	sombrero	idiota
atsegin	jauzi	deleite	salto	huella	principio
atsekabe	egile	pesar	autor	infancia	enseñanza
aukera	orrialde	opción	página	muchacho	umbral
aurrezki	zuritasun	ahorro	blancura	abuela	rueda
azoka	ondorengo	mercado	sucesor	prometida	validez
batuketa	hezur	suma	hueso	sabiduría	consejo
begiratoki	zorabio	mirador	mareo	nieto	portavoz
erruki	apar	piedad	espuma	origen	población
gonbidatu	hiriburu	invitado	capital	garganta	empuje
kontu	gorespen	cautela	exaltación	modelo	anillo
lanbro	onura	bruma	beneficio	sanción	reino
lokarri	iruzur	cordón	engaño	expresión	gestión
lokatz	masail	barro	mejilla	entrada	volcán
norako	eramaile	destino	portador	hermana	taller
oinordeko	nortasun	heredero	personalidad	odio	semana
txanpon	gelditasun	moneda	quietud	camisa	labio
txantxa	iturri	broma	fuente	afición	duda
txerto	abantaila	vacuna	ventaja	suciedad	discusión
txosten	jostailu	informe	juguete	suspiro	olor
ukimen	bidaia	tacto	viaje	conocedor	abandono
aldizkari	arautegi	revista	normativa	frialdad	mina
atseden	atari	descanso	portal	panorama	yema
bizkar	errai	espalda	entraña	luna	pesadilla
burrunba	gerri	zumbido	cintura	mercancía	palacio
errauts	zurruntasun	ceniza	rigidez	madera	fila
euritako	gidari	paraguas	conductor	acción	municipio
gerriko	batasun	cinturón	unidad	saber	formación
gonbidapen	negu	invitación	invierno	oreja	comisión
hildako	etenaldi	fallecido	pausa	estación	denuncia
hizketa	leuntasun	discurso	suavidad	lago	rabo
idazkari	garbitasun	secretario	limpieza	mimbre	asomo
itsasalde	oihal	costa	tela	registro	palabra
itxura	osagarri	aspecto	complemento	mediodía	ocio
jokabide	berezitasun	proceder	singularidad	margen	fibra
kokapen	egiatasun	ubicación	veracidad	longitud	tema
konketa	atezain	lavabo	portero	reproche	fuerza
kontalari	argibide	narrador	explicación	pecho	dicha
kopuru	trebetasun	cuantía	habilidad	mierda	rival
korapilo	erraldoi	nudo	gigante	terremoto	tapa
mutiko	aberastasun	chiquillo	riqueza	variante	censo
ordezkari	berdintasun	delegado	igualdad	compasión	serie
ordoki	gozotasun	llanura	dulzura	costumbre	chica
otsail	argitalpen	febrero	edición	hambre	carga
sukalde	ezaguera	cocina	conocimiento	carbón	burla
txapelketa	ahultasun	campeonato	debilidad	conexión	cara
txistu	erru	silbido	culpa	lentitud	explosión
zenbateko	amildegi	importe	abismo	traductor	velocidad
bukaera	osotasun	desenlace	plenitud	aguja	educación
danbada	ibilbide	estruendo	trayecto	regazo	promoción
edukiera	zailtasun	capacidad	dificultad	vencedor	aversión
edukitze	salbuespen	posesión	excepción	dedo	vigilante
ekoizpen	ezintasun	producción	impotencia	carrera	sillón
erauzketa	ibilaldi	extracción	excursión	nido	osadía
etsipen	suziri	resignación	cohete	liderazgo	quehacer
ezkongai	lasaitasun	soltero	tranquilidad	aparición	hierba
gainbehera	epai	decadencia	veredicto	toma	mando
hizketaldi	ondoez	tertulia	malestar	parada	instinto
hizkuntza	gogortasun	lenguaje	dureza	medición	enero
ikastetxe	gertaera	colegio	suceso	lista	duración
iragazki	ahalegin	filtro	esfuerzo	pantalón	invención
itxaropen	iragarpen	esperanza	anuncio	cerebro	peregrino
jokaera	aditu	actuación	experto	violencia	mesa
kokots	auzitegi	barbilla	tribunal	orina	espina
konponbide	ateraldi	solución	ocurrencia	ombligo	maraña
korridore	arintasun	pasillo	ligereza	molino	entorno
kutxatila	handitasun	estuche	grandeza	edad	hilo
lotsa	epaitegi	vergüenza	juzgado	piel	necesidad
marrazki	betebehar	dibujo	obligación	sacudida	permiso
ordezko	nerabezaro	sustituto	adolescencia	canal	travesía
sukaldari	goraipamen	cocinero	elogio	apoyo	vendedor
txilin	jazarpen	campanilla	persecución	porqué	sudor
aukeraketa	nagusitasun	selección	superioridad	desafío	avenida
baliokide	hurbiltasun	equivalente	cercanía	prioridad	obstáculo
bereizketa	duintasun	separación	dignidad	muerte	manzana
bitxilore	lapurreta	margarita	robo	talla	apellido
ehuneko	ezgaitasun	porcentaje	incapacidad	vientre	maldad
etxebizitza	egonezin	vivienda	inquietud	veneno	hallazgo
ezkontza	urruntasun	matrimonio	lejanía	juego	pastor
ezkortasun	urduritasun	pesimismo	nerviosismo	costado	sala
gatazka	begirune	conflicto	respeto	asiento	semanario
harrokeria	hondamen	vanidad	ruina	bullicio	carnaval
hiruhileko	jarraipen	trimestre	continuación	muralla	comarca
iruzkin	irudi	comentario	imagen	sobrino	vapor
itsustasun	urteurren	fealdad	aniversario	calor	zona
jainkotasun	ipuin	divinidad	cuento	propiedad	accidente
jatetxe	jarraitasun	restaurante	continuidad	compañía	cortina
matxinada	emari	rebelión	caudal	sorpresa	poeta
orrazketa	ipar	peinado	norte	llave	falda
truke	neurritasun	intercambio	moderación	guante	confesor
aurrekontu	afari	presupuesto	cena	hombro	obispo
bizkortasun	ugaritasun	agilidad	abundancia	cerco	bolsillo
hautsontzi	bateratasun	cenicero	convergencia	jaula	cliente
iradokizun	ezabapen	sugerencia	eliminación	corredor	desayuno
konponketa	argitasun	arreglo	claridad	caricia	inventor
ordezkapen	heldutasun	sustitución	madurez	amante	susto
sakontasun	orga	profundidad	carro	salida	viento
suntsidura	zubi	destrucción	puente	recurso	lectura
bitartekotza	hilobi	mediación	tumba	retraso	sede
elkarrizketa	isuri	conversación	flujo	colección	gana
bazkalondo	elur	sobremesa	nieve	occidente	huida
hots	beldur	ruido	miedo	mención	alba
isats	auzi	cola	pleito	huerto	locura
lekuko	izate	testigo	ente	baile	extensión
bazkide	gauerdi	socio	medianoche	cuna	lana
ezkor	zati	pesimista	pedazo	recuerdo	hielo
kezka	euri	preocupación	lluvia	lector	examen
konkor	mihi	bulto	lengua	mezcla	subida
soka	aldi	cuerda	temporada	opresión	cuero
borroka	goiburu	pelea	lema	ramo	cerveza
txinparta	beira	chispa	vidrio	cosecha	juez
barrunbe	zauri	cavidad	herida	escucha	plomo
zoritxar	zoru	desdicha	piso	camino	helada
bizkarralde	orri	respaldo	hoja	carta	llamada
hozkailu	ostegun	nevera	jueves	lunes	nuca
lanbide	argizari	profesión	cera	caza	amigo
zaldizko	apaiz	jinete	cura	deseo	saliva
herrixka	sabai	aldea	techo	posada	sonrisa

**Table A2:** Stimuli used in Experiment 2 using the masked language switching priming paradigm with highly proficient Spanish–English bilinguals.

Prime Words				Target Words	
English		Spanish		Spanish	
Marked	Unmarked	Marked Controls	Unmarked Controls	Marked Set	Unmarked Set
cassock	exhausting	sotana	agotador	sabio	jersey
knock	exchange	golpe	intercambio	bendito	oreja
holy	umbrella	santo	paraguas	potente	piso
lawyer	milestone	abogado	hito	verdadero	pantalón
assembly	flax	montaje	lino	arma	invierno
fodder	plentiful	pienso	abundante	zona	marrón
rubber	shirt	caucho	camisa	ritmo	diente
black	dumb	negro	mudo	espina	triunfo
mockery	storm	burla	tormenta	salud	ceniza
stick	prominence	vara	protagonismo	pandilla	receta
dirty	damaging	sucio	perjudicial	salvaje	mojado
track	recipient	camino	receptor	cirugía	ángulo
term	pupil	plazo	alumno	fusil	esperanza
length	magazine	duración	revista	áspero	guerra
kick	chapel	patada	capilla	tronco	pedazo
gully	receptacle	barranco	recipiente	fundador	humedad
nephew	qualified	sobrino	titulado	espacial	regla
footprint	freedom	huella	libertad	canto	fiebre
sworn	disabled	jurado	impedido	casualidad	lanza
knee	forecast	rodilla	previsión	vivienda	tarifa
madness	prayer	locura	oración	espalda	juventud
knot	limit	nudo	tope	medición	minucioso
kindness	chain	amabilidad	cadena	yate	jugo
fiancée	exit	prometida	salida	bocado	gracioso
enjoyment	unchanging	disfrute	inmutable	agosto	marzo
sweetness	shivering	dulzura	temblor	fraile	susto
address	government	domicilio	gobierno	prado	lana
coldness	foam	frialdad	espuma	marinero	herrero
racket	cheap	bullicio	barato	mejilla	blancura
happy	unexpected	feliz	inesperado	punta	lector
dawn	uprising	alba	revuelta	extranjero	muslo
proximity	stream	cercanía	arroyo	siglo	multa
darkness	shit	oscuridad	mierda	enfermera	campesino
smuggling	chest	contrabando	pecho	martillo	bailarín
upward	lip	ascendente	labio	busca	congreso
privacy	moon	intimidad	luna	lamentable	creencia
safety	surname	seguridad	apellido	disco	sombra
knowledge	lid	saber	tapa	sueldo	cuento
threshold	beard	umbral	barba	vencedor	cocina
breakfast	school	desayuno	escuela	afortunado	pito
sudden	pregnant	repentino	embarazada	conyugal	campana
happiness	beginning	dicha	principio	romero	regazo
opposite	star	contrario	astro	mayo	pelea
thickness	raincoat	espesor	gabardina	mando	hueso
everyday	disgust	cotidiano	asco	cabal	lejanía
newspaper	treatment	periódico	curación	expediente	suavidad
tiredness	church	cansancio	iglesia	paliza	suelo
weakness	mixing	debilidad	mezcla	infierno	nieve
pregnancy	airport	embarazo	aeropuerto	gripe	cuero
barracks	subject	cuartel	asignatura	pequeño	gorro
notebook	deaf	cuaderno	sordo	cima	fantasma
backside	slope	culo	cuesta	osadía	usuario
sickness	unit	mareo	unidad	chica	sorbo
knife	bedroom	cuchillo	dormitorio	cerveza	juzgado
shepherd	harvest	pastor	cosecha	prometido	miedo
strength	speech	fuerza	habla	auto	cerro
pocket	spring	bolsillo	fuente	escopeta	odio
width	stairs	amplitud	escalera	precio	ancho
guilty	hip	culpable	cadera	trimestre	compuesto
checking	painful	revisión	doloroso	semejante	grande
lively	bishop	animado	obispo	sonoro	sacudida
threat	salesman	amenaza	vendedor	asesinato	ducha
apple	edict	manzana	bando	retrete	tela
news	lieutenant	noticia	teniente	crepúsculo	vela
weekly	fishing	semanal	pesca	voluntad	trago
rubbing	discharge	roce	alta	tabla	chorro
navy	ice	armada	hielo	ficha	semana
sword	event	espada	suceso	alquiler	carga
thread	fixed	hilo	fijo	bloqueo	hallazgo
neck	liqueur	cuello	licor	ternura	fundamento
ribbon	smell	cinta	olor	parcela	campamento
wicker	challenge	mimbre	desafío	venta	heredero
mercy	gum	piedad	goma	lavabo	costado
cradle	profit	cuna	beneficio	tesoro	edad
throwing	baroque	lanzamiento	barroco	lujo	poesía
lack	shortage	falta	escasez	hermano	huevo
clock	alleged	reloj	presunto	palabra	carbón
raw	oven	crudo	horno	estanque	millar
sweat	street	sudor	calle	concejal	gitano
joke	rostrum	broma	tribuna	baloncesto	mercado
atrocity	leadership	barbaridad	liderazgo	pueblo	viuda
ability	signature	capacidad	firma	flujo	suciedad
deity	stone	divinidad	piedra	maldito	cambio
pity	disgusting	pena	repugnante	toalla	comercio
neckline	slogan	escote	consigna	siguiente	izquierda
brick	changeable	ladrillo	cambiante	tercio	acta
lazy	spot	vago	mancha	ayudante	esquema
goodness	drum	bondad	tambor	desviación	puerto
cook	portrait	cocinero	retrato	ventana	pastel
hardness	homeland	dureza	patria	último	veloz
official	frost	funcionario	helada	reportaje	recurso
poverty	advisable	miseria	recomendable	grado	llave
quality	glove	calidad	guante	útil	quehacer
beauty	sloping	belleza	inclinado	ladrón	portador
payment	brain	pago	cerebro	mensajero	sierra
similarity	deceased	semejanza	fallecido	ahorro	cirujano
appearance	heart	aspecto	corazón	lectura	fiesta
bucket	exhibition	balde	exposición	necesidad	camarero
sphere	beach	esfera	playa	comarca	edición
ally	husband	aliado	marido	decadencia	orgulloso
spying	jump	espionaje	salto	limpio	teoría
heavy	fleet	pesado	flota	boca	susurro
attack	building	ataque	edificio	blando	bruja
daily	onion	diario	cebolla	codo	tarde
rocket	chin	cohete	barbilla	bienvenida	decorado
effective	leaf	eficaz	hoja	equilibrio	molestia
writing	developing	escritura	revelado	margarita	barro
throw	stop	tirada	parada	incapaz	cierre
midday	advantage	mediodía	ventaja	puente	corredor
effort	iron	esfuerzo	hierro	terremoto	invitado
throat	smile	garganta	sonrisa	compromiso	enseñanza
honesty	invoice	honradez	factura	rueda	raza
equality	depraved	igualdad	vicioso	actual	banda
mummy	misfortune	momia	desdicha	breve	amante
telephone	stable	telefónica	cuadra	resumen	laberinto
approval	rug	aprobación	alfombra	sospechoso	occidente
suggestion	agreement	sugerencia	acuerdo	jinete	tema
package	debt	paquete	deuda	dama	tertulia
loyalty	coin	lealtad	moneda	nube	sombrero
robbery	freezing	robo	helado	cueva	aceite
certainty	uterus	certeza	útero	copa	caza
suffering	unemployed	sufrimiento	parado	motivo	vuelo
physiology	doorman	fisiología	portero	suspiro	mitad
myth	acceptance	mito	aceptación	aficionado	cinturón
symmetry	abrupt	simetría	brusco	occidental	vuelta
emphasis	player	énfasis	jugador	habitual	diversión
pharmacy	fault	farmacia	culpa	éxito	nido
copper	slim	cobre	delgado	taza	canción
nobility	pilgrim	nobleza	peregrino	seco	carta
block	screen	bloque	pantalla	silla	ético
deputy	scales	diputado	balanza	reglamento	pesar
offer	activist	oferta	militante	velocidad	jugada
writer	excitement	escritor	excitación	nacimiento	ligero
needle	shelter	aguja	cobijo	comentario	ateo
birthday	unloading	cumpleaños	descarga	maraña	bruma
drawing	roof	dibujo	techo	molino	gratuita
doubtful	errand	dudoso	recado	carretera	ligereza
support	dancing	apoyo	baile	habilidad	recto
midnight	amazing	medianoche	asombroso	plomo	sequía
unbearable	horn	insoportable	cuerno	maternidad	gramática
workshop	management	taller	gestión	carcajada	anillo
clumsy	goal	torpe	meta	desgaste	cerradura
armchair	blood	sillón	sangre	llamada	principal
unhappy	stamp	desdichado	sello	oriental	comandante
discomfort	lung	malestar	pulmón	calvo	asamblea
viewpoint	forehead	mirador	frente	apertura	altura
stiffness	retirement	rigidez	jubilación	pierna	pila
alms	educated	limosna	culto	pesadilla	sindical
cardboard	stage	cartón	fase	trato	boina
hierarchy	square	jerarquía	plaza	yema	negocio
philosophy	orchard	filosofía	huerto	temblorosa	autobús
abyss	cheese	abismo	queso	ente	informe
